# Acceptability of a community cardiovascular disease prevention programme in Mukono and Buikwe districts in Uganda: a qualitative study

**DOI:** 10.1186/s12889-020-8188-9

**Published:** 2020-01-16

**Authors:** Rawlance Ndejjo, Geofrey Musinguzi, Fred Nuwaha, Rhoda K. Wanyenze, Hilde Bastiaens

**Affiliations:** 1 Department of Disease Control and Environmental Health, School of Public Health, College of Health Sciences, Makerere University, Kampala, Uganda; 2 Department of Primary and Interdisciplinary care, Faculty of Medicine and Health Sciences, University of Antwerp, Antwerp, Belgium

**Keywords:** Acceptability, Cardiovascular disease, Community health workers, Community, Uganda

## Abstract

**Background:**

Cardiovascular diseases (CVDs) are on the rise in many low-and middle-income countries where 80% of related deaths are registered. Community CVD prevention programmes utilizing self-care approaches have shown promise in contributing to population level reduction of risk factors. However, the acceptability of these programmes, which affects their uptake and effectiveness, is unknown including in the sub-Saharan Africa context. This study used the Theoretical Framework of Acceptability to explore the prospective acceptability of a community CVD prevention programme in Mukono and Buikwe districts in Uganda.

**Methods:**

This qualitative descriptive study was conducted in March 2019 among community health workers (CHWs), who would implement the intervention and community members, the intervention recipients, using eight focus group discussions. All discussions were audio-recorded, transcribed verbatim and analysed thematically guided by the theoretical framework.

**Results:**

CHWs and community members reported high eagerness to participate in the programme. Whereas CHWs had implemented similar community programmes and cited health promotion as their role, community members looked forward to health services being brought nearer to them. Although the intervention was preventive in nature, CHWs and community members expressed high interest in treatments for risk factors and were skeptical about the health system capacity to deliver them. CHWs anticipated barriers in mobilising communities who they said sometimes may not be cooperative while community members were concerned about failing to access treatment and support services after screening for risk factors. The major cost to CHWs and community members for engaging in the intervention was time that they would have dedicated to income generating activities and social events though CHWs also had the extra burden of being exemplary. CHWs were confident in their ability to deliver the intervention as prescribed if well trained, supported and supervised, and community members felt that if provided sufficient information and supported by CHWs, they could change their behaviours.

**Conclusions:**

The community CVD prevention programme was highly acceptable among CHWs and community members in Mukono and Buikwe districts of Uganda amidst a few burdens and opportunity costs. Suggestions made by study participants to improve programme effectiveness informed programme design and implementation for impact.

## Background

Cardiovascular diseases (CVDs) are on the rise in many low-and middle-income countries (LMICs) where 80% of related deaths are registered [1, 2]. In these countries, the disease is experienced at younger ages compared to high income countries [3] and risk factors including hypertension and diabetes are increasing. In sub-Saharan Africa, recent systematic reviews have estimated the pooled prevalence of hypertension to range from 24% to over 50% among persons aged 18 years and above [4, 5]. In Uganda, 26.4% of adults are estimated to have hypertension with the highest prevalence in central Uganda (28.5%) which hosts Mukono and Buikwe districts [6]. In Mukono and Buikwe districts, among persons aged 15 years and above, the age standardized prevalence of hypertension is 27.2% [7]. The high CVD burden and risk factors in LMICs are negatively impacting health systems placing a high strain on the scarce health resources and contributing to significant social and economic consequences including loss of economic productivity and high treatment costs negatively affecting household incomes [3, 8].

Community based interventions have shown promise in contributing to population level reduction in the prevalence of CVDs and risk factors [9–12]. This approach involves raising awareness regarding CVDs, encouraging adoption of healthy lifestyles, and early detection and treatment of risk factors in line with self-care interventions, a key strategy to deal with non-communicable diseases (NCDs) and advance Universal Health Coverage [13]. Self-care interventions among communities are key since most LMICs do not have the capacity to implement large scale chronic disease programmes for CVDs in healthcare facilities [14–16]. The current shortage of health workers in many LMICs have made CHW programmes a necessity and these have registered several benefits for communicable diseases prevention and control [17, 18]. Uganda launched its CHW programme in 2001 with Village Health Teams charged with the responsibility of empowering communities to take control of their own health and wellbeing, and actively participate in the management of local health services [19]. In the Ugandan context, CHWs are volunteers with the ability to read and write in the local language, selected by their communities and serve as a link between them and health facilities [19, 20]. With the shifting disease burden in Uganda like in many LMICs, attempts are being made to empower CHWs and communities for roles in the prevention and control of NCDs. However, the extent to which community programmes for NCD prevention are acceptable remain largely underexplored amidst the increasing focus on effectiveness studies.

Sekhon et al. (2017) defined acceptability of healthcare interventions as a multifaceted construct that reflects the extent to which people delivering or receiving a healthcare intervention consider it appropriate, based on anticipated or experienced cognitive and emotional responses to the intervention [21]. The need to assess acceptability of interventions from the perspectives of intervention deliverers and recipients is increasingly becoming important in health care and public health research as this has a huge bearing on intervention implementation and uptake, and subsequent effectiveness and sustainability [22]. Acceptability can be assessed at three levels: prospectively – before intervention enrollment, concurrently – while the intervention is being implemented and retrospectively - following implementation of an intervention [21]. In this study, we applied the Theoretical Framework of Acceptability [21] to prospectively explore the acceptability of a community CVD prevention programme in Mukono and Buikwe districts in Uganda so as to inform the proposed intervention and enhance its uptake.

### Description of intervention

The community CVD programme includes working with CHWs, existing networks and community structures to promote knowledge, improved lifestyles, risk assessment and cardiovascular health as previously described [23]. The programme involves training of CHWs to conduct health education, promote lifestyle change through motivational interviewing and goal setting techniques, screen for risk factors at the community using non laboratory tools, and conduct house-to-house visits within their communities. Other community networks and structures are also empowered to promote healthy lifestyle behaviours, screening for risk factors and cardiovascular health. Information, education and communication materials including in electronic and print forms are circulated to enhance health promotion and improved lifestyle behaviours.

## Methods

### Study area

This study was conducted in rural and sub-urban communities in Mukono and Buikwe districts in the central region of Uganda approximately 35.0 km and 57.4 km from Kampala, the country’s capital, respectively. These districts, with a population of 1,000,000 people [24] of whom over 70% reside in rural areas, are strategically located between the two biggest urban areas of Jinja and Kampala. The districts are situated along the main transport corridor connecting Kampala, Uganda’s capital through Nairobi to Mombasa Sea Port in Kenya. The major economic activities in these districts include small scale agriculture, fishing and small-scale businesses.

### Study design and population

This was a qualitative descriptive study using the Theoretical Framework of Acceptability as a guiding theory. Data were collected through focus group discussions (FGDs) among CHWs and community members in four selected parishes of Kyabakadde and Nabalanga in Mukono district, and Busabaga and Lugala in Buikwe district. These parishes were selected because they were scheduled to benefit from a CVD prevention programme implemented under the SPICES – Scaling up Packages of Interventions for Cardiovascular disease prevention in selected sites in Europe and sub-Saharan Africa – project [23]. In each of the parishes, four villages that had been randomly selected to receive the community intervention had their CHWs invited to participate in the FGDs. These FGDs consisted of both male and female CHWs of varying ages. The Uganda Ministry of Health guidelines recommend each village to be served by four to five CHWs dependent on its size, each responsible for about 30 households. Community FGDs were also convened in each of these parishes homogenous for age (25 to 40 years and 41 to 70 years) and sex (males and females) forming four groups to allow free discussion among peers with similar characteristics. This sample was considered sufficient and all data were collected and analysed. FGDs provided an opportunity for discussion and further probing resulting into a nuanced understanding of how forces in the community may influence effective intervention implementation [25]. Moreover, compared to interviews, FGDs allow collective brainstorming of ideas, issues and solutions creating a “synergistic group effect” [26]. All study participants were approached face to face by a team of CHWs who invited them for the discussions and all invited participants accepted to participate in the discussion.

### Data collection

Data collection took a period of one week in March 2019 prior to intervention implementation. CHWs and community member groups were convened within the community for the discussions. A FGD guide (See Additional files 1 and 2) developed basing on the Theoretical Framework of Acceptability [21] was used during the discussions. The Framework conceptualizes acceptability as a multifaceted construct with seven components (Table 1).

**Table 1:** Theoretical Framework of Acceptability constructs

Construct	Definition
Ethicality	Extent to which the intervention has good fit with an individual’s value system
Affective attitude	How an individual feels about the intervention;
Intervention coherence	Extent to which the participant understands the intervention and how it works
Burden	Perceived amount of effort required to participate in the intervention
Opportunity costs	Extent to which benefits, profits, or values must be given up to engage in the intervention.
Self-efficacy	Participant’s confidence that they can perform the behaviour(s) required to participate in the intervention
Perceived effectiveness	Extent to which the intervention is perceived to be likely to achieve its purpose

The guide, which was pretested in a community with similar characteristics, was structured into introductory sections with greetings and rapport building questions preceding a thorough description of the proposed intervention and series of questions on acceptability of the intervention. The questions were open ended and organised from general to specific ones with probes. Data on socio-demographic characteristics of participants including age, sex and education level were also collected at the end of the discussion. Research Assistants who were graduates with experience in conducting qualitative research working as a pair of males and females moderated the discussions. One Research Assistant asked questions and directed the conversation while another audio recorded the proceedings and took notes. The discussions which lasted an hour on average took place in *Luganda*, the mostly spoken local language of the area. Some of the Research Assistants had worked with the CHWs during a mapping and listing exercise and the baseline survey undertaken by the project prior to intervention implementation. The research assistants were supervised by a member of the research team (RN) throughout the data collection process.

### Data management and analysis

All audio-recordings from the discussions were transcribed verbatim and concurrently translated into English by the experienced Research Assistants with expertise in both languages. Another Research Assistant who attended the interview read through the transcript to ensure that the activity had been done adequately. A theoretical thematic analysis method based on the theoretical framework guided the analysis following the semantic approach [27]. Specifically, RN and GM read a sample of the transcripts identifying meanings and patterns across the data and independently developed a codebook for analysis which they later discussed, unified and organized based on the theoretical framework. All transcripts were exported into Atlas ti version 6.0.15 to guide the coding process where text was coded using the codebook with any new codes fitted accordingly. Afterwards, the codes were re-examined and re-categorized by RN and GM where similar codes were related to form sub themes under the framework constructs. Selected quotations supporting codes have been presented to supplement the study findings. Reporting for the study has been guided by the Consolidated Criteria for Reporting Qualitative Research guidelines [28] (See Additional file 3).

## Results

A total of 41 CHWs participated in the four FGDs, 28 of whom were female. Among CHWs, average age was 46 years (range 25 to 65) while 24 had primary education. Most (35) of the CHWs were subsistence farmers, and had served their communities for an average of 12 years (range 1 to 20). For community members, a total of 37 with an average age of 39 years (range 25 to 70) participated in the four FGDs, half (19) of whom were female. Most (19) community members had primary education and 8 in 10 were subsistence farmers (Table 2).

**Table 2:** Characteristics of study participants

	CHWs (*n* = 41)	Community members (*n* = 37)
Characteristic	Number	Number
Parish		
Busabaga	13	12
Kyabakadde	11	7
Nabalanga	9	10
Lugala	8	8
Sex		
Female	28	19
Male	13	18
Age (years)		
25 to 40	14	22
41 to 70	27	15
Education level		
None	0	2
Primary	24	19
Secondary	17	16
Occupation		
Farmer	35	32
Business	4	3
Mechanic	1	0
Housewife	1	0
Student	0	2
Years working as CHW		
1 to 5	7	–
6 to 10	9	–
Above 10	25	–

CHWs and community members reported never to have received CVD prevention programmes in their communities except for Nabalanga parish where some CHWs had had a short training to measure blood pressure among their members conducted by a non-governmental organisation. The study findings are organised under the seven themes of the Theoretical Framework of Acceptability: ethicality, affective attitude, burden, opportunity costs, perceived effectiveness, self-efficacy and intervention coherence. Table 3 summarizes the study themes, sub-themes and codes.

**Table 3:** Acceptability of a community CVD programme among CHWs and community members based on the Theoretical Framework of Acceptability (TFA) framework

TFA construct / theme	Sub-theme	Summary codes	
		CHW	Community
Ethicality	Programme fits community value system	• Belief in seeking healthcare from facilities. • Interest in remaining healthy. • No contradiction with religious or political views.	• Similar approaches have been employed • No contradiction with religious, political or cultural views. • Intervention elements such as education and counseling are acceptable
Affective attitude	Willingness to engage in intervention	• Participation in similar community based programmes. • Community health services delivery is our role. • Appointed and trusted by the community members. • High perceived disease burden • High need for CVD services • Opportunity to widen scope of work.	• High perceived CVD burden. • Interest in screening and treatment services. • Opportunity to know CVD status. • Difficult to access care at health facilities.
	Conditions for intervention participation	• Well trained to carry out assigned tasks. • Obtain all costs related to intervention delivery. • Regular refresher trainings. • Honesty in terms of services.	• Well trained and motivated CHWs • Early information about planned events. • Continuous involvement in project activities. • Honesty in terms of services.
	Appropriateness of intervention delivery strategies (group gatherings and house to house visits)	Group gatherings • Has high reach to community members. • Varrying confidence in conducting group sensitizations. House to house visits • Flexible timing. • Quality time with community members. • Reach the underserved.	• Appropriate with high reach among community members. House to house visits • Reach the underserved.
Intervention coherence	Understanding of intervention	• Training in CVD prevention and control. • CHWs educating community members. • Assess lifestyle risk factors and provide advice. • Encourage screening for risk factors. • Offer treatment to community members.	• CVD prevention programme. • Promotion of healthy lifestyles. • CHWs trained to sensitise community. • Community members mobilising others.
Burden	Reaching communities	• Transportation, big areas of coverage, meals and equipment • Being exemplary • Carrying heavy screening equipment.	
	Mobilising communities	• High interest of in financial gains • Resistant groups like youths • Uncooperative community members • Uncommon lifestyle choices • Fatigue due to repetitive information. • Unavailability at homes	• Low and inconsistent turn up • Unavailability at homes • Unwillingness to change • Unreceptivity to CHWs • Other duties and responsibilities
	Community health work related challenges	• Inadequate training for proposed roles. • Transport and weather challenges. • Low motivation and incentives. • Inadequate tools and materials.	• Negative community attitudes towards CHWs. • CHWs not being exemplary.
	Health system barriers	• Unprepared health workers. • Lack of required equipment and drugs. • Poor relationship with health workers. • Low belief in health system.	• Lack of transport to distant health facilities. • Lack of medicines at health facilities. • Lack of screening equipment. • Absence of health workers at facilities. • Low belief in health system.
Opportunity costs	Reduced time for other activities	• Reduced time for attending to farmlands, domestic work, socializing and travelling away.	• Reduced time for income generating activities and tending to farms.
	Benefits to communities	• Access to treatment. • Healthy members contributing to their communities. • Savings due to prevention of CVDs. • Knowing CVD status.	• Obtaining treatment and screening for CVDs. • Knowing CVD status early. • Healthy community members. • Knowledge on CVDs and risk factors.
	Benefits to CHWs	• Information to keep healthy and better manage CVDs. • Community members acknowledging CHW expertise. • Fame and earning community trust.	
Self-efficacy	Confidence to deliver intervention or change behaviour	• Previous experience in community programmes. • Anticipated adequate training. • Positive and eager community for information.	• Increased and empowerment to contribute to behavior change • Information sharing among community members.
Perceived effectiveness	Intervention is effective	• Timely and consistent information key to reduce unhealthy behaviours • CHWs serving as examples	
	Measures to increase effectiveness	• Utilise community resources (community, religious and cultural leaders and community radio / loudspeakers) • Increase functionality of the health system • Involve external persons in intervention delivery • Provide information education and communication materials such as leaflets in the local language.	

### Ethicality

Both CHWs and community members stated that the proposed CVD programme was well within their community’s values, norms and beliefs. CHWs emphasized that in their communities, most people believed in seeking health care from health facilities and were interested in keeping healthy and thus the intervention complemented their beliefs. They also noted that the programme was not divisive and “*does not in any way conflict with our religious values or political views”* (FGD3, CHW4, female, 41 to 70 years) and thus would be accepted by the community and themselves.

*“In our community, majority of people believe in healthcare provided through established health facilities. Even though there are a handful who consult traditional practitioners, the bulk accesses care from health facilities which you will often find crowded with people. These will be interested in the proposed programme.”* (FGD 1, CHW 11, male, 41 to 70 years).

Moreover, the community stated that the intervention was using similar approaches that had been employed by other organisations and did not conflict with their culture.

*“This programme fits well within the value system of the community and I don’t think there is anyone who can resist it.”* (FGD 4, CM 2, female, 41 to 70 years).

In one of the groups, a community member stated that:

*“In this village, I have never heard of any group that refuses health care however, there is a small group in another village that we hear does not accept medicines and drugs. Fortunately, this intervention involves more of education and counseling to support people to abandon unhealthy behaviors which I think they will accept.”* (FGD 2, CM 7, male, 25 to 40 years).

### Affective attitude

CHWs positively perceived the proposed intervention and expressed willingness to participate in its delivery. They had implemented similar community based programmes and community health services delivery being their role, they perceived the intervention as an opportunity to widen their scope of work. Additionally, CHWs noted that they had been appointed by the community itself and thus were trusted, and there was a huge need for CVD services in their community.

*“I am very happy because you are elevating us to another level. We were known for treating malaria and supplying mosquito nets but today when I get back to my community, I will tell them how I am going to educate them about CVDs including what they ought to eat and do. They will be very pleased and pay attention to know about another prevention intervention. It is true that people in our communities are suffering from such diseases.”* (FGD 2, CHW 2, female, 25 to 40 years).

When asked about how the community would receive the intervention, most CHWs said that community members were happy to utilise health interventions if well explained to them and prefer that services are extended nearer to them. Indeed, in the community FGDs, members noted that the CHW-led intervention would be positively received within the community due to the perceived high CVD burden, lack of access to screening and treatment services, and usual difficulties in accessing health care at health facilities. Moreover, the community would have an opportunity to screen for CVDs and know their status and thus would be willing to participate in the programme.

*“I am glad this programme is coming because many people have not been aware of their CVD health status. Recently during your [SPICES Project] baseline survey, we noticed that almost all villages had some cases of these conditions. In other words, it is like you people knew what was ailing our community. You may find that if I don’t have such a disease, my wife or child is having it or someone else. Therefore, it will be valuable when that programme comes into our community.”* (FGD 1, CM 5, female, 41 to 70 years).

Amidst stating their willingness to participate in the intervention, in their FGDs, CHWs were keen to set conditions for leading the intervention delivery within communities. They strongly suggested the need to be well trained to carry out assigned tasks and build their confidence in addition to accessing regular refresher trainings. Moreover, community members pointed out that properly trained CHWs would increase acceptance of the programme.

*“We expect that you will give us an adequate training to make us comprehend well what we are supposed to do. You may plan to train us in one session and presume that we shall understand everything there and then but our brains are elderly and are not like yours, the young people who grasp things so fast. With inadequate training, we shall not be able to deliver what you want us to do but if you engage us in numerous trainings, we shall be able to pick up slowly and make people understand what we have been taught.”* (FGD 1, CHW 5, female, 25 to 40 years).

The community members expressed the need to be informed of any planned events early enough to enable them plan their participation, and continuous involvement in project activities. Both CHWs and community members noted that they expected honesty from the project in terms of services it would offer, and a functional health system with trained health workers and facilities stocked with drugs.

Regarding appropriateness of the proposed strategies of intervention delivery, most CHWs preferred carrying out sensitizations in large group gatherings such as places of worship, community meetings and savings groups as this would enable them reach out to many people at ago. This opinion was also shared by most community members. However, some CHWs noted reservations in terms of their confidence to carry out sensitizations to large groups.

*“To emphasize, we are known and acknowledged in villages and worship places thus we can access both churches and mosques. Therefore if we have the confident voices, we will be accepted and these are appropriate platforms for us.”* (FGD 2, CHW 5, female, 41 to 70 years).

On the other hand, there was consensus among CHWs that moving door-to-door was appropriate and would allow them to deliver the intervention during their flexible times, have quality time with family members and reach community members who rarely converged in public places. Community members too suggested that door-to-door sensitisations would reach those who are usually left out or underserved.

*“I think they [CHWs] should move door to door for the community to benefit from the programme because in most cases, people do not get to know about such programmes even when they are sick. For instance, there are people who stay far away and are burdened to come to the health facility. So, I think we should do this house-to-house visit for each village.”* (FGD 3, CM 7, male, 41 to 70 years).

### Intervention coherence

CHWs and community members both expressed a good understanding of the CVD prevention programme and described it well when called upon. Some of the CHWs even rehearsed how they would make the move to explain the programme to community members.

*“When I get to your house, I would first introduce myself for you to know me if you don’t already. Then, I would inform you about the main reason that has brought me about how we can prevent CVDs. I then seek permission to ask you some questions. If you accept, I also take the body measurements. Using the findings, I can then advise you. In case you drink a lot of alcohol, eat a lot of fried and fatty foods which we perceive to be good, I advise you to change the behavior to start cooking boiled food without oils to prevent blockage of your blood vessels and eat some vegetables too.”* (FGD 2, CHW 8, female, 41 to 70 years).

However, some CHWs did not appreciate how the intervention would work without giving treatment or drugs saying *“medicines are going to be given to me as the CHW, so that I can attend to the patients within the community before sending them to the health facility”* (FGD 1, CHW 1, female, 41 to 70 years) *or “it is now our turn to treat high blood pressure and CVDs”* (FGD 4, CHW 5, female, 25 to 40 years). Some aspects of the intervention were also difficult for the CHWs to comprehend including the use of non-laboratory tools to screen community members although some described it well stating that *“I will be able to assess and know if the community member has been having unhealthy diet, drinking a lot of alcohol among others and advise accordingly”* (FGD 2, CHW 8, female, 41 to 70 years) and motivational interviewing.

For community members, the programme was about “*preventing CVDs and if you accept to change your lifestyle as advised it will be of benefit to you”* (FGD 4, CM 2, female, 41 to 70 years) *and* CHWs *“will be trained to sensitise us and share CVD information”* (FGD 3, CM 6, male, 40 years)*.* Community members also voiced eagerness to mobilise and share information with their colleagues, though some expressed doubt in the project materializing that “*are you certain that the CHWs will come on ground to educate people like you said? What exactly should we tell the community? We might tell them about the intervention and they eagerly wait for it and then you never come back*”. (FGD 2, CM 8, male, 25 to 40 years).

### Burden

CHWs mentioned challenges related to intervention delivery such as transport and big areas of coverage to be burdensome stating that “*It is very hard to walk these big villages but if there is some transport, work can be made easier.”* (FGD 1, CHW 12, male, 41 to 70 years)*.* Other burdens were attributed to: lack of meals while in the field and inputs such as equipment, education materials and reporting forms. The CHWs also expressed concern that although positive, participating in the intervention would infer on them a responsibility of living exemplary lives especially in terms of CVD prevention for community members to learn from which are part of the sacrifices they needed to make.

*“For me, I will abandon being in community and sensitizing people that eating a lot of salt is bad and that adding it to sauce when raw is what brings heart diseases and then I do the opposite. I will not be the person who goes and drinks [alcohol] and at the same time forbids others from drinking that it is one of the risk factors for developing heart diseases. So, if we have been using some things which are not good for the heart, we have to give them up and be able to serve as good examples to the rest of the community.”* (FGD 1, CHW 5, female, 25 to 40 years).

CHWs also highlighted that sometimes it is hard to mobilise community members for services especially with increasing interest in financial gains and that other community groups like youths *“are challenging to approach and easily resist what is being told to them than the older people some of whom have these conditions and easily listen”.* (FGD 2, CHW 6, female, 25 to 40 years). Furthermore, they stated that community members may be easily fatigued with repetitive information and it would be much harder to have communities change their behaviours especially to adopt those that have not been part of their ways such as jogging as a form of physical activity as the community is active in other ways and so they needed extra support.

*“People will have to change from their former mindset and lifestyles as advised. For example eating certain vegetables that they do not know or have, reducing oil and fat consumption and reducing alcohol intake for example one who has been taking five bottles of alcohol gradually reduces with time. This will however not be that easy for some members.”* (FGD 2, CHW 3, female, 41 to 70 years).

The other barriers anticipated by CHWs were: inadequate training for proposed roles, transport and weather challenges, low motivation and incentives, lack of adequate tools and materials for their work. Regarding the door-to-door strategy, CHWs noted that they would be faced with uncooperative community members who would not welcome them in their homes. Indeed, in one of the community groups, a participant noted that *“sometimes a CHW visits a household and a community member hides from them”* (FGD 2, CM 9, male, 25 to 40 years). Moreover, there was also a preparedness among CHWs that not all community members would be receptive to information to change their behaviours as *“in society, there is always that one person that no matter how much effort you put in, they will completely refuse to change even in our own households”.* (FGD2, CHW 10, female, 41 to 70 years). The other CHW concerns were: not finding community members at home especially during the peak planting season which the community members agreed with, that *“many members especially those involved in agriculture at a large scale keep in their gardens almost all day in the sowing season and so CHWs won’t find them at home.”* (FGD2, CM 1, male, 25 to 40 years)*, and* provided screening equipment being too heavy to carry while moving door to door. CHWs also highlighted burdens from the community side and these were: low and inconsistent turn up for events, unavailability of community members at homes, unwillingness to change behavior and being engaged in other duties and responsibilities to attend or engage with CHWs.

Relatedly, community members expressed concern about the intervention deliverers stating that community attitudes towards CHWs were not always positive due to dissatisfaction with previous projects and some not being exemplary and so the community members may not take the intervention keenly to change their behaviours.

*“The problem we have with CHWs is that we fully know their behaviour; some of them come advising us to change certain behaviours yet we know that they too are culprits of the same behaviours and diseases. This makes it hard for one to accept the advice they give. Therefore, it will require you [SPICES project] to first train and educate CHWs to be good examples to other community members so that we carry on the advice they give.”* (FGD 2, CM1, female, 41 to 70 years).

In all CHW FGDs, the biggest barrier highlighted was as an unprepared health system where health workers are not ready to receive referred community members. They mentioned lack of required equipment and drugs where community members who are encouraged to visit health facilities leave without receiving services and the poor relationship with health workers where sometimes they may not take seriously persons referred by a CHW. They felt these challenges would affect community morale and participation in the intervention.

*“The challenge that we usually face after we tell community members what they are suffering from and refer them to the health facility is that they find that there are no drugs there. So people just waste their time. It also makes you lose hope because you took your time doing your role and they [health facility] really don’t care. So we usually sacrifice our time spent in the community walking for nothing yet we are not even rewarded anything. At least it would be better if we walk through our communities and get some results.”* (FGD 4, CHW 7, male, 41 to 70 years).

Relatedly, community members anticipated barriers in terms of access to health services including the lack of transport to distant health facilities, cost of medicines and their unavailability at health facilities, lack of screening equipment and the absence of health workers at facilities which affected confidence in the health system. Community members strongly expressed concern about the possibility of getting screened to know their status and not being able to access treatment or other support services from the project or health facility. Although this was expressed by all community groups, it was far more pronounced in the 40 to 70-year-old women group.

*“You people can sensitize us about all these diseases but you might leave it at that and not give us treatment. I might go and advocate for the project in my village because most of us have high blood pressure and diabetes as you know our ages, but shall we get treatment?”* (FGD 4, CM 5, female, 41 to 70 years).

### Opportunity costs

Among CHWs, the most commonly mentioned cost for participating in implementing the intervention was reduced time for other activities especially for attending to farmlands, domestic work and other works to accommodate the CVD programme needs. Others mentioned that they would also have to reduce time invested in socializing and travelling away from their communities in order to deliver on the proposed tasks.

*“There will be reduction in time allocated to activities that we have been doing. If I was finishing my work by midday, I will have to adjust and finish by 10am. This is because I have to first go back home and finish up the domestic work before I start moving to households.”* (FGD 2, CHW 4, female, 41 to 70 years).

Similarly, community members stated that their participation in intervention activities would affect the time they spend running their businesses and tending to their farms. This they said was the major sacrifice for them and some would spare time to engage in intervention activities especially if informed in advance.

*“I am a woman running a salon and mobile money businesses and I will lose my customers when I come to participate in the intervention. However, I am ready to make some sacrifices at the time when you call us to gather if informed in time.”* (FGD 1, CM 13, female, 25 to 40 years).

CHWs anticipated some benefits and expressed a strong need for motivation through financial incentives such as salary and allowances and non-financial incentives including branded T-shirts for identification purposes, bags to carry paper forms and equipment, and protective wear such as umbrellas and gumboots for use during rainy seasons. The expectation of financial incentives was also supported by some community members who said that CHWs sacrifice their time to serve them and so should be well facilitated.

*“CHWs sometimes devote themselves to do community work but every so often are failed by transport yet they also give up some work where they could have earned money and only get paid small emoluments. It is possible that a CHW will not devote his/her time to do community work without earning money for their families which is something that should be looked into.”* (FGD 3, CM 2, male, 25 to 40 years).

Among the benefits to communities, CHWs strongly stressed the need for treatment being made available either within their communities or at the health facility. This too was the most highly anticipated benefit among community members in all FGD groups as some said they were nursing related conditions and did not receive much help from health facilities and thus looked forward to receiving drugs from CHWs while others wanted to be screened for CVD within their communities.

*“In the villages, we have many patients especially for hypertension but the problem is long distances from our villages to the health facility and not finding drugs there. With your support, we should have drugs delivered to CHWs at the village level rather than the health facility to bring services closer to community members.”* (FGD 3, CM 2, male, 25 to 40 years).

The CHWs also expressed that the intervention would ensure that their members are healthy, able to contribute to their communities and obtain associated benefits such as savings due to preventing diseases.

*“I want to put this intervention into action because when my community is healthy, I am also happy. There are some community developmental activities which bring us together but when you visit some members to implement these, you find that they are not well which also affects my morale of working in the community. So, I am going to support it [intervention] because it is going to restore health of our community people.”* (FGD 1, CHW 4, male, 25 to 40 years).

The anticipated benefits among community members were: obtaining knowledge and advice regarding CVDs and healthy lifestyles, getting to know their CVD status early, obtaining treatment for their CVDs, and achieving a healthy community. Similarly, CHWs were interested in obtaining information to keep healthy and others to better manage their conditions. Other CHWs looked forward to communities further acknowledging their expertise, becoming famous and earning trust.

### Self-efficacy

CHWs were confident in their ability to deliver the intervention and achieve the desired results. They attributed their confidence to previous experience supporting community programmes, the anticipation of being appropriately trained and equipped, the receptiveness of community members to health interventions and eagerness to take on new information.

*“Personally, before I was taught [HIV] counseling, I did not know how to do it and that it was remedy on its own. Later, I got to learn that counseling is another way of comforting someone. Therefore, as a CHW, I feel that I can deliver the intervention well; I can educate very well and carry out measurements but now on this issue of CVDs, what I lack is getting trained because they [community] will ask me some things which I should have responses for.”* (FGD 2, CHW 7, male, 41 to 70 years).

Similarly, most community members in addition to stating that CHWs would deliver the intervention if trained, believed in their power to change and adopt new behaviours once provided with more information, empowered and receive adequate support from the CHWs. They were also eager to share information with others to increase their awareness and influence them.

*For me, I say that we definitely ought to change because in most cases, people do certain things out of ignorance of risk factors but after knowing the danger in them, they would definitely change. I am confident and I will follow the advice of the CHWs and adopt the suggested behaviours for instance not to eat certain foods which are too fatty or oily.* (FGD 3, CM 3, male, 25 to 40 years).

### Perceived intervention effectiveness

CHWs expressed their belief in the intervention achieving its intended goal. They noted that with information, community members would most likely adopt healthy behaviors which would contribute to CVD prevention and control.

“*If you help them realize their risk, some might think twice and change their lifestyles depending on your advice. For one who has been taking five bottles of alcohol, they will then take four and gradually reduce with time.”* (FGD 2, CHW 10, female, 41 to 70 years).

Some CHWs also said that with them adopting healthy behaviours it would be easier for the community members to do so. For the community, they agreed that with time and consistency of information, change would occur gradually.

*“I think and strongly believe that change will be there because personal minds go on changing when someone keeps on getting new ideas and information. If education sessions are provided well and in plenty, and the CHWs are trained to the extent that you have answers for most questions asked by community members, people’s minds will be changing as regards CVD prevention and control.”* (FGD 3, CM 1, female, 41 to 70 years).

CHWs went ahead to suggest measures to increase effectiveness of intervention implementation within the community and suggested that wider community resources should be employed including community, religious and cultural leaders and community radio or loudspeakers for community mobilisation. The use of community groups such as savings groups, organised clusters, village meetings and gatherings such as religious ones were highlighted as ways to reach masses of people at a given time.

*“We are requesting that you involve local leaders in helping to mobilise community members for the intervention. These can mobilise a large group of people which we can’t do as CHWs. The people are familiar with us but if we involve leaders like the local council one chairpersons, area councillors and other politicians, they can be listened to more.”* (FGD 3, CHW 1, female, 41 to 70 years).

These suggestions also came out prominently within community groups where they in addition requested for village coordinators and community champions to increase engagement and the use of village clusters where they existed to reach them. Community members also suggested that: outreaches further be made to their communities to extend services such as blood pressure screening closer to them and a specific day set aside for project activities within the community.

A functional health system was another factor prominently mentioned by both CHWs and community members. CHWs stated that *“if I refer a community member to the health facility and the health workers attend to her well and give her treatment, it will cause an assurance in others to trust and believe in what we say more.”* (FGD 1, CHW 3, female, 25 to 40 years) Corroboratively, community members said, *“if a person comes to the health facility and gets tested and receives free treatment, they will go back and tell their neighbour, even the second person would tell the third, and so forth.*” (FGD 1, CM 3, female, 25 to 40 years) The other element of the functional health system mentioned by CHWs was availability of necessary screening equipment and respect and recognition by health workers.

Another suggestion from CHWs was the need to have external persons such as project staff and health workers attend community events and accompany them in delivering the intervention as this would be better received by the community. This was reiterated by the community members who also suggested that involvement of the project team in community activities would be key and would increase the community’s faith in the CHWs because *“some people can only accept the advice given if they see some experts working hand in hand with CHWs*” (FGD 2, CM 3, male, 25 to 40 years). This view was most prominent among the 25 to 40-year-old males in the semi-urban community group who also said that CHWs should continually be encouraged to be exemplary.

*“In addition to what was said by one of the participants, CHWs are good but they can only be seen to be good if you [project team] come along with them in communities than them coming alone. For example you can decide to move alongside CHWs in the communities every Tuesday to people’s households and you educate them, which will make sense. This is better than sending them alone. My suggestion is that you could first come along with the selected CHWs so that community members see you around.”* (FGD 2, CM 2, male, 25 to 40 years).

*One of the CHWs stated that:*

*We also urge you [project team] to be present when we are sensitizing people because they tend to listen more to those they do not know than we, the common figures. In case the Chairman calls a meeting, you people from SPICES [the project] should also come to attend and introduce us like, “we are the people from SPICES and these are CHWs whom you appointed and we have come to work with them to deal with different conditions”.”* (FGD 1, CHW 5, female, 25 to 40 years).

CHWs also suggested that extra information ought to be provided to community members in form of information education and communication materials such as leaflets in the local language which they can give out after providing them with information.

## Discussion

The SPICES project planned to implement a community CVD programme in Mukono and Buikwe districts in Uganda. Prior to the intervention, we explored the acceptability of the programme among CHWs and potential service recipients using the Theoretical Framework of Acceptability [21] to inform its adaptation. Assessing prospective acceptability provided an opportunity to highlight intervention aspects that could be modified to enhance uptake and impact.

This study revealed that the intervention was highly acceptable to both CHWs and community members. Indeed, CHWs were eager to participate in the CVD prevention programme owing to it being their health promotion role and the desire to serve their communities as advanced in previous studies [29–31]. Such virtues have been strong motivators of community health work [32]. However, CHWs needed to sacrifice crucial productive time and social networks in addition to meeting community’s expectations to change their behaviours to implement the intervention. How the CHWs balance these priorities with their health service roles continues to be crucial and could determine how acceptable the intervention would be to them. For community members, they looked at the intervention programme as an opportunity to obtain knowledge about CVDs and their prevention, and access extended screening and treatment services within their communities or health facility. Similar aspirations and benefits have been reported in other community programmes [33, 34].

The burdens and opportunity costs reported by CHWs and community members are all in line with previous Ugandan studies [29–31, 35] and most probably shaped by previous programmes. The CHW programme in Uganda is voluntary where CHWs are expected to offer their time to implement health activities for the benefit of their communities without pay [19, 20] and this should explain their expectations and demands as most facilitation and incentives (financial and non-financial) are provided by partner organisations. Previous research in Uganda has demonstrated that CHWs valued non-financial incentives more than the financial ones [29, 30]. Moreover, provision of financial incentives sometimes erodes trust between CHWs and their communities and stakeholders, and when not well managed affects the their morale [36]. However, the World Health Organization guideline on health policy and system support to optimise community health worker programmes recommends providing practising CHWs with a financial package in line with assigned duties, training, roles and working hours [37].

Although community members were at times skeptical of CHWs, they still desired that CHWs are provided with screening equipment and drugs to ease their access of these services at the village level. Previous Ugandan studies have found that amidst the lack of trust in CHWs, communities still have high expectations of them to provide more diagnostic and curative services [38, 39]. This could portray the value the community attaches to biomedical interventions. Indeed, research has shown that CHWs who give drugs or commodities to community members are more respected [36, 40]. Norms and community perceptions of illness could also play a part in the high interest in biomedical interventions. For example, a study in Iganga and Mayuge districts of Uganda reported that communities viewed lifestyle risk factors such as unhealthy diets as indicative of wealth and a high social standing and stated that changing these would tantamount to sacrifice of the ‘good life’ [41]. Such perceptions could explain the increasing interest in drugs to address conditions that the community prefers to believe are external to their lifestyle [41]. Communities should be supported to regain the belief in preventive measures and their ability to prevent diseases, maintain health and cope with illness or disability as stipulated in the World Health Organization consolidated guideline on self-care interventions for health [13]. This is especially key in dealing with NCDs that mostly require lifestyle changes. Since trust and maintenance of confidentiality are key to CHWs’ acceptance in delivering health services [38, 42, 43], CHWs trainings, support and supervision should cover effective ways to relate and improve their relationships with community members and the health system, which is as important as services delivery [31, 38, 42].

The role of a functional health system came out prominently in the study and the potential ways in which it affects the work of CHWs was featured. Previous research has shown that community members are not eager to engage with a health system that does not cater for their needs and easily lose trust in it [35, 38] and the CHWs [36, 39] thus affecting their health seeking behaviours. The unpreparedness of primary health care facilities to support NCD prevention and control has been reported previously [15, 16, 35]. Patients have also complained about the quality of care and unavailability of commodities and supplies [35] while CHWs are weary of their relationship with health workers at facilities [42]. These factors could also offer an explanation for the high demand for biomedical interventions from CHWs. Cognizant of these gaps, the design of the overall project intervention incorporated a health facility intervention to build the capacity of health workers at the primary health care and higher levels to better prevent and manage NCDs [23]. The health worker training focuses on equipping them with better communication skills and improving the patient - provider and provider - CHW relationships. Basic equipment to support diagnosis and monitoring of CVDs is provided in selected health facilities in addition to lobbying the Ministry of Health to ensure NCD drugs availability at facilities. These activities are implemented prior to the community intervention so that health facilities act as a referral mechanism for community members along the health system. With a functional health system, CHWs are more motivated and less stressed in doing their work and community members may adopt better health seeking behaviours [36, 43] important for CVDs prevention and control accruing benefits to the population.

Implementation of the community programme involves thorough training of CHWs to build their confidence and capacity for CVD prevention and control. The project team offers frequent support supervision visits to CHWs to support intervention implementation and regular meetings with CHWs are convened to discuss and share experiences among themselves. A systematic review in China noted that high quality training and supervision of CHWs were key facilitators of their NCD prevention and control roles [43]. Once in a while, the project team and health workers join CHWs during community sessions and offer any support while endorsing their work. Moreover, a study done in a rural context in Eastern Uganda reported that accompaniment by supervisors improved acceptance of CHWs by the community [38] and in a Kenyan study, CHWs requested for shadowing while at work by their supervisors to provide them feedback [39]. As part of the intervention, CHWs conduct door-to-door sensitisations and screen community members for CVD using non-laboratory interheart tools and refer those with high risk scores to health facilities. The programme capitalizes on all community resources as suggested by the study participants to reach out to all community members to encourage them to adopt healthy lifestyles for CVD prevention. Indeed, several community institutions including churches, markets, schools and local organisations, and avenues such as community gatherings, health facility outreaches, clusters, local and religious leaders and savings groups are used to reach community members. The programme provides non-financial incentives including branded t-shirts, umbrellas and gumboots for identification and motivation purposes of CHWs taking part in the intervention, and costs related to intervention delivery such as transport and communication are met.

### Study limitations and strengths

This study did not capture perspectives of other stakeholders such as local leaders and health workers as these had been explored during the situational analysis phase of the project and their suggestions informed the proposed community CVD prevention programme [44]. Two of the female community member focus group discussions were moderated by males which may have affected expressions of community members. Broadly, the fact that CHWs would implement the intervention and they and community members expecting some benefits from the programme could have influenced their responses and perceived acceptability. On the other hand, involvement of both CHWs and community members provided an opportunity for triangulation of responses increasing their validity. Moreover, the use of the Theoretical Framework of Acceptability also ensured that all critical dimensions of acceptability were explored and considered in intervention design and implementation increasing potential for successful intervention implementation, its uptake, effectiveness and sustainability. The study findings have potential to inform design and implementation of related community health programmes in similar contexts.

## Conclusion

The community CVD prevention programme was highly acceptable among CHWs and community members in Mukono and Buikwe districts of Uganda. Indeed, the intervention was in line with community values and beliefs, appropriate and understood by CHWs and community who were confident to implement it and/or change their behaviours. Usual burdens affecting community health work and similar opportunity costs were reported amidst the expectation of the intervention being effective. Community CVD prevention programmes should embed sufficient training for CHWs with supportive supervision systems to build their capacity to earn community trust and effectively deliver interventions. The role of a functional health system in ensuring appropriate linkages and the acceptance and uptake of community interventions was underscored and the need to empower communities to regain their belief in preventive over biomedical interventions is key. In community interventions, the use of available community resources should be prioritised and all stakeholders brought on board to make their contributions. The perspectives of CHWs and community members informed intervention design and implementation to be evaluated in a real-world setting using implementation science approaches.

## Supplementary information

**Additional file 1.** Focus group discussion guide for community health workers

**Additional file 2.** Focus group discussion guide for community members

**Additional file 3.** Completed COREQ checklist

## Data Availability

The data/transcripts used during the current study are available from the corresponding author on reasonable request.

## Abbreviations

CHWs: Community Health Workers
CM: Community Member
CVD: Cardiovascular disease
FGD: Focus Group Discussion
LMICs: Low and Middle Income Countries
NCDs: Non-Communicable Disease
SPICES: Scaling up Packages of Interventions for Cardiovascular disease prevention in selected sites in Europe and sub-Saharan Africa;
