# Supplementary material for: Acceptability of a community cardiovascular disease prevention programme in Mukono and Buikwe districts in Uganda: a qualitative study
Source: BMC Public Health. 2020 Jan 16;20:75. doi: 10.1186/s12889-020-8188-9 (PMC6966788; doi:10.1186/s12889-020-8188-9)
Supplement: Supplementary file 1 — Additional file 1. Focus group discussion guide for community health workers [file 12889_2020_8188_MOESM1_ESM.docx]

**Acceptability of a community cardiovascular disease prevention programme in Mukono and Buikwe districts in Uganda: a qualitative study**

### **Focus group discussion guide for community health workers**

**General information**

District: ___________________________ Sub county: _____________________________

Parish: ____________________________ Village: ________________________________

Date: _____________________________ Name of note taker: _______________________

FGD number: ________________ Number of FGD participants: ________________________

Starting time: ________________________ End time: _______________________________

**Brief instructions for the FGD facilitator/moderator:**

**Instructions to interviewer:**

- This discussion guide shouldn’t be followed word-for-word like a questionnaire, rather it should guide your discussion with the participants and ensure that all topics are covered.
- These interviews should be open-ended, with the participants’ responses determining the direction of the discussion. Be flexible. The discussion is expected to last about an hour.
- Start by building rapport with respondents, introductions and seek written consent for the discussion from each participant. Also discuss taking notes; seek oral consent for using recorder.
- Set and agree on ground rules.
- Record number assigned to each CHW, age, sex, education level and duration in CHW work at the end.

**Questions**

1. Are there any CVD prevention programs or activities in this community? Which ones are they?
   - How do people feel about current CVD prevention programs or activities? Do they engage in these programmes or activities?
   - Do any of such programmes involve CHWs? If so, what activities do the CHWs participate in? Are there any barriers that you face in carrying out these activities?
   - To what extent do current CVD prevention programs meet existing needs?

***To strengthen CVD prevention efforts within the community, Makerere University School of Public Health through the SPICES project together with the Ministry of Health and Mukono and Buikwe district local governments are proposing to implement a CVD prevention program in your community. The project will train community health workers (CHWs) to:***

- *Conduct health education for CVD prevention.*
- *Screen for risk factors using an assessment tool and measuring blood pressure, weight, height and hip and waist.*
- *Provide motivational advise for behavioural change.*

*This is going to be achieved through:*

- *House to house visits.*
- *Group meetings.*
- *One on one counseling.*

1. How essential is this intervention to meet the needs of community members you serve as a CHW? How will the intervention fill current gaps?
2. Do you think the interventions (health education, screening at household level, and advise) would be acceptable to: the CHWs? Community members? Local, religious and cultural leaders? Health workers?
3. What about the strategies (house to house visits, group meetings and counseling) to achieve the proposed interventions? Are these acceptable?
4. Does this intervention fit well within your value system? If so, to what extent? If not, what doesn’t fit well?
5. How do you feel about this intervention? Are you looking forward to implementing it? Why?
6. How burdensome will carrying out this intervention be to you? In what ways?
7. Are there things (such as work, other responsibilities, benefits, profits or values) that you will have to give up to carry out this intervention? To what extent would you have to do this?
8. Do you think this intervention is likely to achieve its purpose of encouraging community members to change behavior and reduce their risk of CVD?
9. Are you confident that you will cope with delivery of the intervention? Why?
10. In your view, how would you explain this intervention and how do you think it works?
11. What are your views on the appropriateness of the proposed intervention? Do you think the interventions can be delivered at the community level?
    - Probes: Culture, community interest, political environment, community structures.
12. What current support such as personnel, equipment, resources, supplies do you have as CHWs to deliver CVD prevention services in the community?
13. As a CHW, do you think you can routinely carry out these tasks (health education, screening and advise)?
14. What do you foresee as hindrance to project implementation? community participation? What can be done to address the anticipated hindraces?
15. What opportunities and resources are available within the community to facilitate implementation?
16. Do you think the proposed intervention is likely to be sustainable? Why?

- What modifications or adaptations will need to be made to sustain the initiative over time?

***Thank you for your time***
