# Supplementary material for: Acceptability of a community cardiovascular disease prevention programme in Mukono and Buikwe districts in Uganda: a qualitative study
Source: BMC Public Health. 2020 Jan 16;20:75. doi: 10.1186/s12889-020-8188-9 (PMC6966788; doi:10.1186/s12889-020-8188-9)
Supplement: Supplementary file 2 — Additional file 2. Focus group discussion guide for community members [file 12889_2020_8188_MOESM2_ESM.docx]

**Acceptability of a community cardiovascular disease prevention programme in Mukono and Buikwe districts in Uganda: a qualitative study**

### **Focus group discussion guide for community members**

**General information**

District: ____________________________ Sub county: ______________________________

Parish: ______________________________ Village: ________________________________

Date: ______________________________ Name of note taker: _______________________

FGD number: ________________ Number of FGD participants: ________________________

Starting time: ________________________ End time: _______________________________

**Brief instructions for the FGD facilitator/moderator:**

**Instructions to interviewer:**

- This discussion guide shouldn’t be followed word-for-word like a questionnaire, rather it should guide your discussion with the participants and ensure that all topics are covered.
- These interviews should be open-ended, with the participants’ responses determining the direction of the discussion. Be flexible. The discussion is expected to last about an hour.
- Start by building rapport with respondents, introductions and seek written consent for the discussion from each participant. Also discuss taking notes; seek oral consent for using recorder.
- Set and agree on ground rules.
- Record number assigned to each community member and their age, sex and education level at the end.

**Questions**

1. Are there any CVD prevention programs or activities in this community? Which ones are they?
   - How do people feel about current CVD prevention programs/practices/processes?
   - Do any of such programmes involve CHWs? If so, what activities do the CHWs participate in?
   - To what extent do current CVD prevention programs meet your needs?

***To strengthen CVD prevention efforts within the community, Makerere University School of Public Health through the SPICES project together with the Ministry of Health and Mukono and Buikwe district local governments are proposing to implement a CVD prevention program in your community. The project will train community health workers (CHWs) to conduct health education, behavioural counselling for key CVD risk factors, and referrals to the health facility for high risk individuals. We expect that the CHWs will move door to door but also utilize community gatherings to educate community members on key preventive measures for CVDs. They will also use a form to assess an individual’s risk and refer high risk individuals to the health facility.***

1. How essential is this intervention to meet your needs as community members?
   - Is it acceptable to you as community members? Why or why not?
   - How will the intervention fill current gaps?
2. Does this intervention fit well within your value system? If so, to what extent? If not, what doesn’t fit well?
3. How do you feel about this intervention? Are you looking forward to taking part? Why?
4. How burdensome will participation in the intervention be to you? In what ways?
5. Are there things (such as work, other responsibilities, benefits, profits or values) that you will have to give up to participate in the intervention? To what extent would you have to do this?
6. Do you think this intervention is likely to achieve its purpose of encouraging people to change behavior and reduce their risk of CVD?
7. Are you confident that you will adopt the suggested behaviors? Why?
8. In your view, how would you explain this intervention and how do you think it works?
9. Comment about the appropriateness of the intervention? Do you think it can be delivered in the community?
   - Why or why not?
10. What community infrastructure exists to support delivery of the intervention? (E.g. regular community gatherings?, local leadership)
11. Do you feel you can participate in the project?
12. What benefits do you foresee or how will the intervention meet your needs? (improved access to information and support, reduced need to go to the health facilities etc.)
13. How do you think other community members will respond to the intervention?
14. What barriers might hinder you from fully participating in the intervention? (e.g. (time?) What can be done to reduce these barriers?
15. What opportunities are available to facilitate implementation of the project? (receptive community? Other community resources? Etc)
16. Do you think the proposed intervention is likely to be sustainable? Why? What are likely modifications or adaptations that need to be made to sustain the initiative over time?

***Thank you for your time***
